# A meta-analytic evaluation of sex differences in meningococcal disease incidence rates in 10 countries

**DOI:** 10.1017/S0950268820002356

**Published:** 2020-10-02

**Authors:** Manfred S. Green, Naama Schwartz, Victoria Peer

**Affiliations:** School of Public Health, University of Haifa, Haifa, Israel

**Keywords:** meningococcal disease, sex differences, incidence rate ratios, meta-analysis, male predominance

## Abstract

The magnitude and consistency of the sex differences in meningococcal disease incidence rates (IR) have not been systematically examined in different age groups, countries and time periods. We obtained national data on meningococcal disease IR by sex, age group and time period, from 10 countries. We used meta-analytic methods to combine the male to female incidence rate ratios (IRRs) by country and year for each age group. Meta-regression analysis was used to assess the contribution of age, country and time period to the variation in the IRRs. The pooled male to female IRRs (with 95% CI) for ages 0–1, 1–4, 5–9, 10–14 and 15–44, were 1.25 (1.19–1.32), 1.24 (1.20–1.29), 1.13 (1.07–1.20), 1.21 (1.13–1.29) and 1.15 (1.10–1.21), respectively. In the age groups 45−64 and over 65, the IR were lower in males with IRRs of 0.83 (0.78–0.88) and 0.64 (0.60–0.69), respectively. Sensitivity analysis and meta-regression confirmed that the results were robust. The excess meningococcal IR in young males and the higher rates in females at older ages were consistent in all countries, except the Czech Republic. While behavioural factors could explain some of the sex differences in the older age groups, the excess rates in very young males suggest that genetic and hormonal differences could be important.

## Introduction

Invasive meningococcal disease, caused by Gram-negative bacteria *Neisseria meningitidis*, is a contagious disease that can result in severe morbidity, high case fatality and serious sequelae [1, 2]. The disease is endemic in most countries and is periodically responsible for large epidemics [2]. Serogroups A, B, C, W and Y are responsible for most cases of invasive disease. In the ‘meningitis belt’ in Africa, immunisation against group A meningococcus was introduced in 2010 [3] and some countries in Europe routinely immunise infants against group C meningococcus using the conjugate vaccine [4]. Serogroup B is now the commonest strain across Europe (https://assets.publishing.service.gov.uk/government/uploads/system/uploads/attachment_data/file/842368/hpr3819_IMD-ann.pdf) and only recently has an effective vaccine become available [5]. At any one time, about 10% of the population carry meningococcal bacteria in the nasopharynx without experiencing the disease [6, 7]. The mechanism of the infection has not fully been established.

Sex differences in the incidence rates (IR) of a variety of infectious diseases have been documented [8–10]. There is evidence that the IR for meningococcal disease differ between the sexes, based on population-based studies, case series and case−control studies [11–15]. In addition, in several studies case-fatality rates were higher among females [16–18]. On the other hand, female infants appear to develop a stronger immune response to group C meningococcal vaccine [19].

To the best of our knowledge, the consistency of sex differences in meningococcal IR by age, over different populations and for prolonged periods of time have not been reported. In this study we used national data from a number of countries to address this issue. The findings could yield important insights to the mechanisms of meningococcal disease.

## Methods

### Source of data

In order to ensure the reliability of the results we identified official sources that provide detailed information on meningococcal disease at the national level over a number of years. We searched for data from all countries in Europe, American and Australasian continents (including New Zealand), in which reporting of meningococcal disease by age and sex is compulsory and available through official websites or representatives of the national institutes. All countries for which we obtained data at the resolution of age and sex were included in the analyses. We identified 10 countries from different continents as follows: Europe (Czech Republic, England, Finland, Germany, Poland and Spain), Australasia (Australia and New Zealand), North America (Canada) and Asia (Israel). Data from Australia for 2001−2016 were obtained from the National Notifiable Diseases Surveillance System (NNDSS) [20], for Canada for 1991−2015 from the Canadian Notifiable Disease Surveillance System (CNDSS) [21], for the Czech Republic, for 2008−2013, from the Institute of Health Information and Statistics [22], for England, for 1990−2016, directly from Public Health England (PHE) representative, for Finland, from the National Institute for Health and Welfare (THL) [23], for Germany, for 2000−2015, from the German Federal Health Monitoring System [24], for Israel, for 1998−2016, from the Department of Epidemiology in the Ministry of Health, for New Zealand, for 1997−2015, from the Institute of Environmental Science and Research (ESR) [25], for Poland, for 2006−2016, from the National Institute of Public Health [26], and for Spain for 2005−2015, from the Spanish Epidemiological Surveillance Network [27]. Information about the population size by age, sex and year for the Australian population was obtained from the Australian Bureau of Statistics [28], for Canada from Statistics Canada [29], for the Czech Republic from the Czech Statistical Office [30], for England, from the Office for National Statistics [31], for Finland from the Statistics Finland's PX-Web databases [32], for Germany, from the German Federal Health Monitoring System [33], for Israel from the Central Bureau of Statistics [34], for New Zealand from Stats NZ, Infoshare [35], for Poland from official web site Statistics Poland [36] and for Spain from the Demographic Statistics Database [37].

### Ethical considerations

National, open access, aggregated and anonymous data were used and thus there was no need for approval from the University of Haifa ethics committee.

### Statistical analyses

We calculated annual IR by sex and age group, for each country, between years 1990 and 2016 and divided the entire study period into three or four year intervals: 1990−1993, 1994−1997, 1998−2001, 2002−2005, 2006−2009, 2010−2013 and 2014−2016 (the last period included two or three years). An attempt was made to analyse the data for equal time periods (for some countries data during certain periods were available for single years). IR by sex (per 100,000) and age were calculated using the number of reported cases divided by the population size (by sex) and multiplied by 100,000. The age groups investigated were <1 (infants), 1–4 (early childhood), 5–9 (late childhood), 10–14 (puberty), 15–44 or 15–39 (young adulthood), 45–64 or 40–59 (middle adulthood) and 65 + /60 + (senior adulthood) years. The information reported from Canada, England, Finland and New Zealand refers to the same age group as other countries except for the following: 15–39, 40–59 and 60+. Australia and Finland do not report disaggregated data for ages <1 and 1–4 but for age 0–4 and were thus excluded from the analyses in that age group. The male to female IRR was calculated by dividing the annual IR in males by that of females, by age group, country of origin and time period.

As in our previous studies, we used meta-analytic methods to pool the IRRs over time periods and countries [10, 38, 39]. The meta-analyses were carried out using STATA software version 12.1 (Stata Corp., College Station, TX). The outcome variable was the male to female IRR. Pooled IRR's for each age group were computed by combining all countries and time periods together. Cochran's *Q* statistic was used to analyse for heterogeneity. Tau^2^ and *I*^2^ estimates were used for the evaluation of the variation between studies. These statistics were used to assist in deciding on the use of the fixed or random effects model. If significant heterogeneity was observed (if *I*^2^ ⩾ 50% and/or the *Q* test yielded a *P*-value <0.1), the random effects model [40] was used to estimate pooled IRRs and 95% confidence intervals (CI). Otherwise, the fixed effects model was considered. Since the power of the tests for heterogeneity is low, we generally used the more conservative random effects model.

Egger's test (sensitivity analysis) was performed to evaluate the extent of impact of each individual ‘'study’ on the pooled estimate. The pooled meningococcal IRR was computed after omitting one country or one time period at a time. In order to assess whether there were countries or time periods that are outliers by sample size, we used the methods for estimation of the possibility of publication bias. In order to explore the contributions of age group, country and time periods to the heterogeneity of the IRRs, we carried out meta-regression analyses.

## Results

Data on the incidence of meningococcal disease by age group and sex, over a number of years, were obtained from 10 countries. Male and female meningococcal disease IR (per 100,000 populations) in all countries for each age group and year groups are presented in Table 1.

**Table 1.** Details of the countries included in the study, by sex and age group – total cases (n - cumulative total of cases for given years), population size (N - cumulative total of the population for given years), IR per 100 000 and IRR.

			Males		Females		
Age	Country	Years	n/N	IR	n/N	IR	IRR
<1	Canada	1991–2015	442/4682619	9.4	345/4446799	7.8	1.2
	Czech Republic	2008–2013	32/349195	9.2	32/332712	9.6	1.0
	England	1990–2016	2403/8725051	27.5	1771/8306732	21.3	1.3
	Germany	2001–2016	591/5740478	10.3	461/5448550	8.5	1.2
	Israel	1998–2016	131/1486100	8.8	92/1410400	6.5	1.4
	New Zealand	1997–2015	538/576900	93.3	372/548520	67.8	1.4
	Poland	2006–2016	302/2177523	13.9	264/2055764	12.8	1.1
	Spain	2005–2015	618/2679186	23.1	483/2514548	19.2	1.2
1–4	Canada	1991–2015	520/19156418	2.7	380/18225737	2.1	1.3
	Czech Republic	2008–2013	48/1410748	3.4	49/1343670	3.6	0.9
	England	1990–2016	3600/34821935	10.3	2686/33207057	8.1	1.3
	Germany	2001–2016	899/23509315	3.8	2686/22311030	3.2	1.2
	Israel	1998–2016	156/5731500	2.7	111/5443300	2.0	1.4
	New Zealand	1997–2015	876/2308880	37.9	643/2191980	29.3	1.3
	Poland	2006–2016	424/8763810	4.8	324/8294052	3.9	1.2
	Spain	2005–2015	886/10880587	8.1	750/10233932	7.3	1.1
5–9	Australia	2001–2016	201/11398585	1.8	156/10814642	1.4	1.3
	Canada	1991–2015	160/24668602	0.6	150/23469919	0.6	1.0
	Czech Republic	2008–2013	15/1532669	1.0	15/1450621	1.0	1.0
	England	1990–2016	1104/42989082	2.6	925/41012194	2.3	1.1
	Finland	1995–2016	18/3440956	0.5	14/3297629	0.4	1.3
	Germany	2001–2016	266/30760941	0.9	241/29187252	0.8	1.1
	Israel	1998–2016	98/6616300	1.5	58/6287700	0.9	1.7
	New Zealand	1997–2015	325/2899540	11.2	263/2752910	9.6	1.2
	Poland	2006–2016	111/10753278	1.0	115/10206501	1.1	0.9
	Spain	2005–2015	335/13017097	2.6	272/12287011	2.2	1.2
10–14	Australia	2001–2016	155/11377822	1.4	112/10797396	1.0	1.4
	Canada	1991–2015	210/25685783	0.8	175/24391864	0.7	1.1
	Czech Republic	2008–2013	8/1416001	0.6	13/1339518	1.0	0.6
	England	1990–2016	646/42597565	1.5	505/40624659	1.2	1.3
	Finland	1995–2016	25/3522497	0.7	11/3375446	0.3	2.3
	Germany	2001–2016	253/33455166	0.8	240/31724889	0.8	1.0
	Israel	1998–2016	40/6106400	0.7	32/5807300	0.6	1.2
	New Zealand	1997–2015	275/2919850	9.4	178/2776650	6.4	1.5
	Poland	2006–2016	93/11130177	0.8	73/10591951	0.7	1.1
	Spain	2005–2015	158/12301238	1.3	119/11627137	1.0	1.3
15–39/15–44	Australia	2001–2016	1118/73591102	1.5	1014/72741755	1.4	1.1
	Canada	1991–2015	1024/143987472	0.7	898/140453550	0.6	1.2
	Czech Republic	2008–2013	78/13725818	0.6	79/12978912	0.6	1.0
	England	1990–2016	1471/234901126	0.6	1318/233399206	0.6	1.0
	Finland	1995–2016	217/18898064	1.1	121/18050351	0.7	1.6
	Germany	2001–2016	1606/257895408	0.6	1242/247590330	0.5	1.2
	Israel	1998–2016	96/29586200	0.3	71/29264100	0.2	1.5
	New Zealand	1997–2015	687/13546700	5.1	658/13976900	4.7	1.1
	Poland	2006–2016	494/92802239	0.5	338/90097352	0.4	1.3
	Spain	2005–2015	746/110542308	0.7	708/105413400	0.7	1.0
40–59/45–64	Australia	2001–2016	253/41988401	0.6	302/42573071	0.7	0.9
	Canada	1991–2015	311/110461323	0.3	386/109655649	0.4	0.8
	Czech Republic	2008–2013	10/8403729	0.1	22/8624880	0.3	0.3
	England	1990–2016	417/175100277	0.2	488/177644620	0.3	0.7
	Finland	1995–2016	67/16513241	0.4	96/16307550	0.6	0.7
	Germany	2001–2016	301/181698132	0.2	362/181849520	0.2	1.0
	Israel	1998–2016	23/12368500	0.2	27/13327000	0.2	1.0
	New Zealand	1997–2015	147/10201030	1.4	191/10685350	1.8	0.8
	Poland	2006–2016	114/55127862	0.2	140/59180678	0.2	1.0
	Spain	2005–2015	263/63103755	0.4	328/64340310	0.5	0.8
60+/65+	Australia	2001–2016	122/21417772	0.6	218/25538457	0.9	0.7
	Canada	1991–2015	232/64590224	0.4	433/78346403	0.6	0.7
	Czech Republic	2008–2013	6/4087584	0.1	8/5999018	0.1	1.0
	England	1990–2016	263/129663953	0.2	595/163257756	0.4	0.5
	Finland	1995–2016	37/11159619	0.3	80/15066114	0.5	0.6
	Germany	2001–2016	206/108019284	0.2	426/149862231	0.3	0.7
	Israel	1998–2016	11/5759300	0.2	16/7566600	0.2	1.0
	New Zealand	1997–2015	67/6302700	1.1	104/7386000	1.4	0.8
	Poland	2006–2016	24/23115840	0.1	69/37363573	0.2	0.5
	Spain	2005–2015	197/37127234	0.5	401/49879431	0.8	0.6

The IR between the countries varied widely. The highest IR in both sexes in all age groups were observed in New Zealand. Between 1991 and 2011, there were large epidemics of meningococcal disease in New Zealand that appears to explain the particularly high IR [41, 42]. Overall, sex-specific rates by age were highest in males for infants and in 1–4-year-olds. There was a decrease in the IR of meningococcal disease in adults, in both groups of males and females.

It should be noted that England immunises routinely against meningococcus C at three months, Germany at 12–23 months and Spain at four months.

**Fig. 1.**
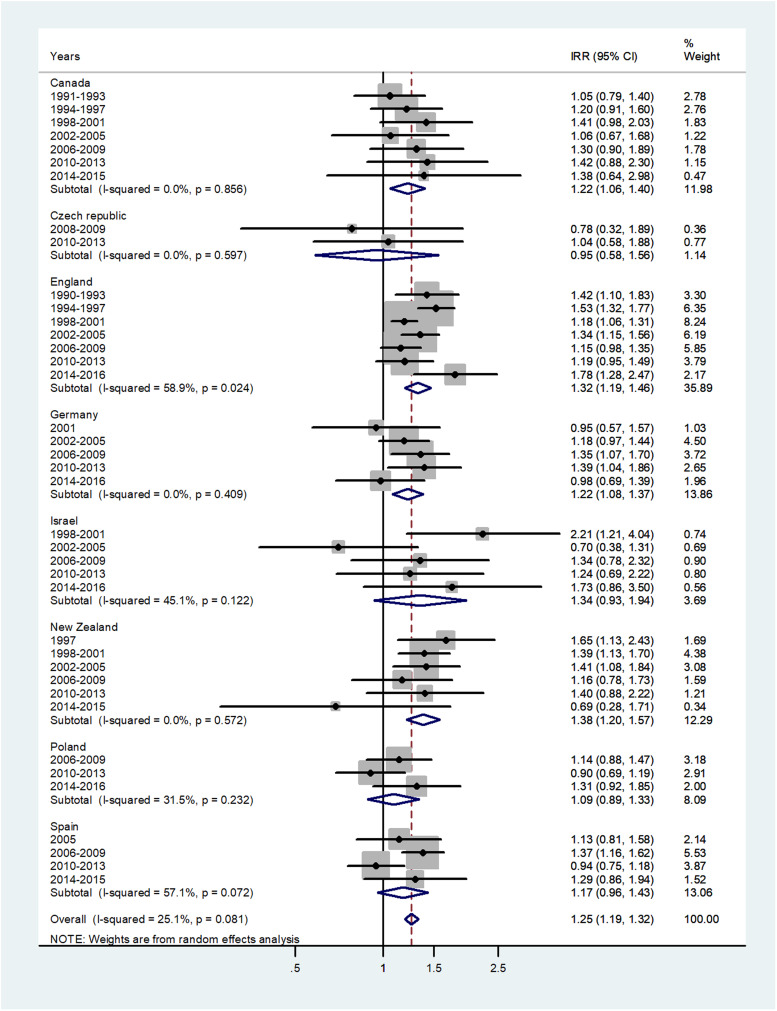
Forest plot of the male to female meningococcal disease IRR in infants (<1) for eight countries by time period. CI = 95% confidence interval.

The results of the meta-analyses are presented as forest plots. The forest plot for infants is presented in Figure 1. The overall male to female IRR in infants (age <1) was 1.25 (95% CI 1.19–1.32) with *I*^2^ = 25.1%. The IRR varied from 0.95 in Czech Republic to 1.38 in New Zealand.

**Fig. 2.**
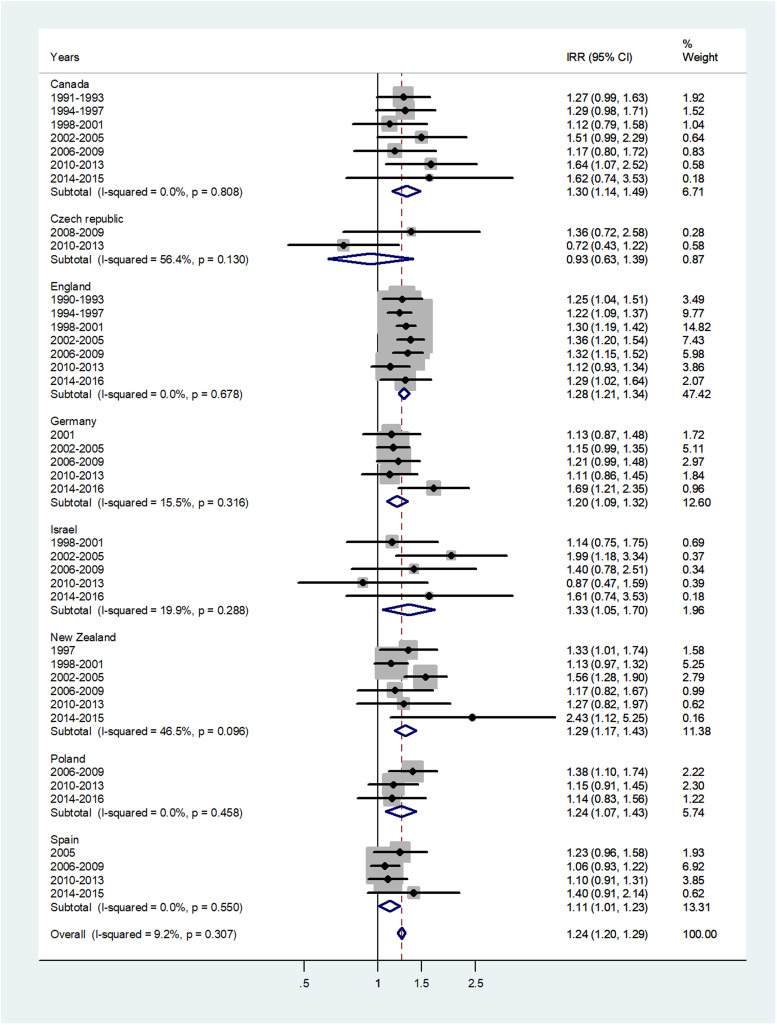
Forest plot of the male to female meningococcal disease IRR for children at age 1–4 years, for eight countries by time period. CI = 95% confidence interval.

The forest plot for age 1–4 is given in Figure 2. The overall IRR = 1.24 (95% CI 1.20–1.29) with *I*^2^ = 9.2% and varied from 0.93 in Czech Republic to 1.33 in Israel.

**Fig. 3.**
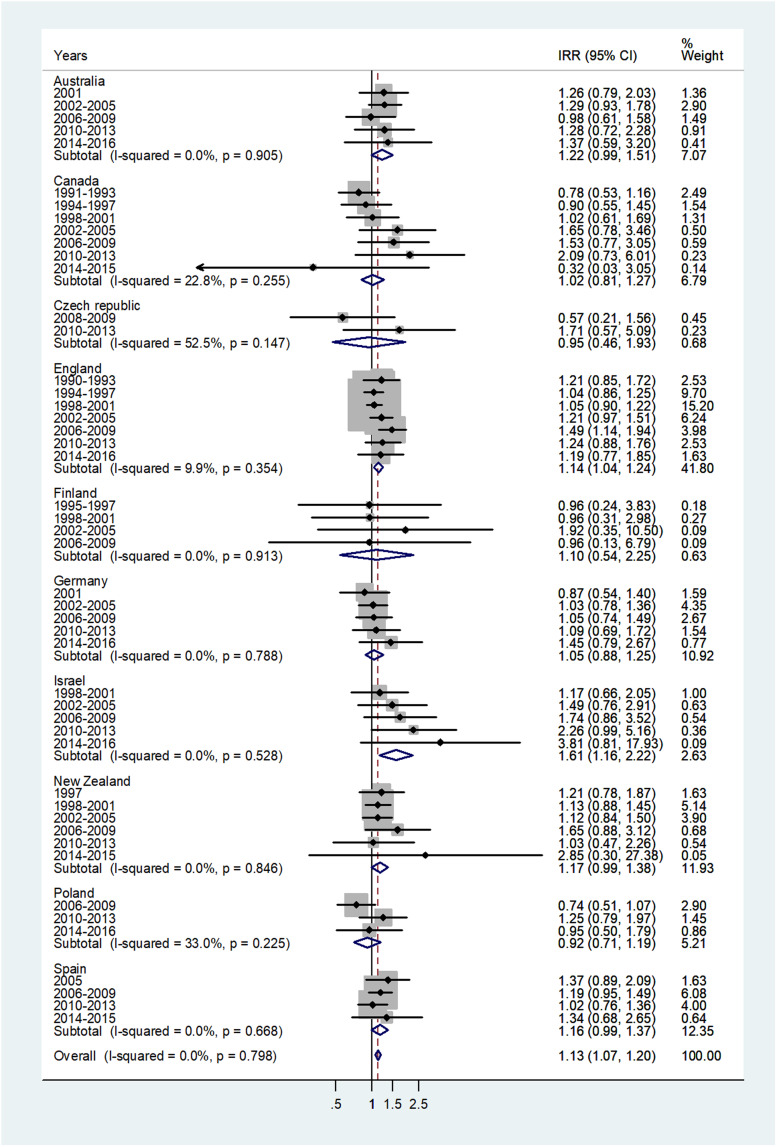
Forest plot of the male to female meningococcal disease IRR for children at age 5–9 years, for 10 countries by time period. CI = 95% confidence interval.

The forest plot for age 5–9 is given in Figure 3. The overall IRR was 1.13 (95% CI 1.07–1.20) with (*I*^2^ = 0%) and varied from 0.92 in Poland to 1.61 in Israel.

**Fig. 4.**
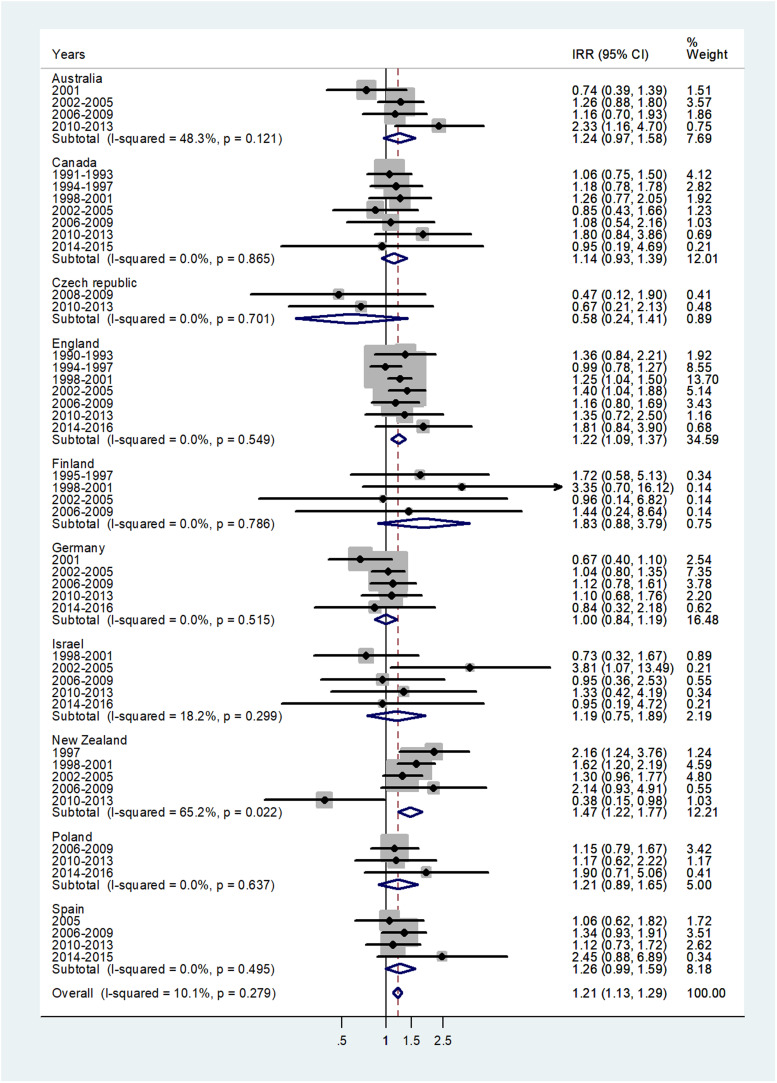
Forest plot of the male to female meningococcal disease IRR for children at age 10–14 years, for 10 countries by time period. CI = 95% confidence interval.

The forest plot for age 10–14 is shown in Figure 4. The overall IRR was 1.21 (95% CI 1.13–1.29) with (*I*^2^ = 10.1%) and varied from 0.58 in Czech Republic to 1.83 in Finland.

**Fig. 5.**
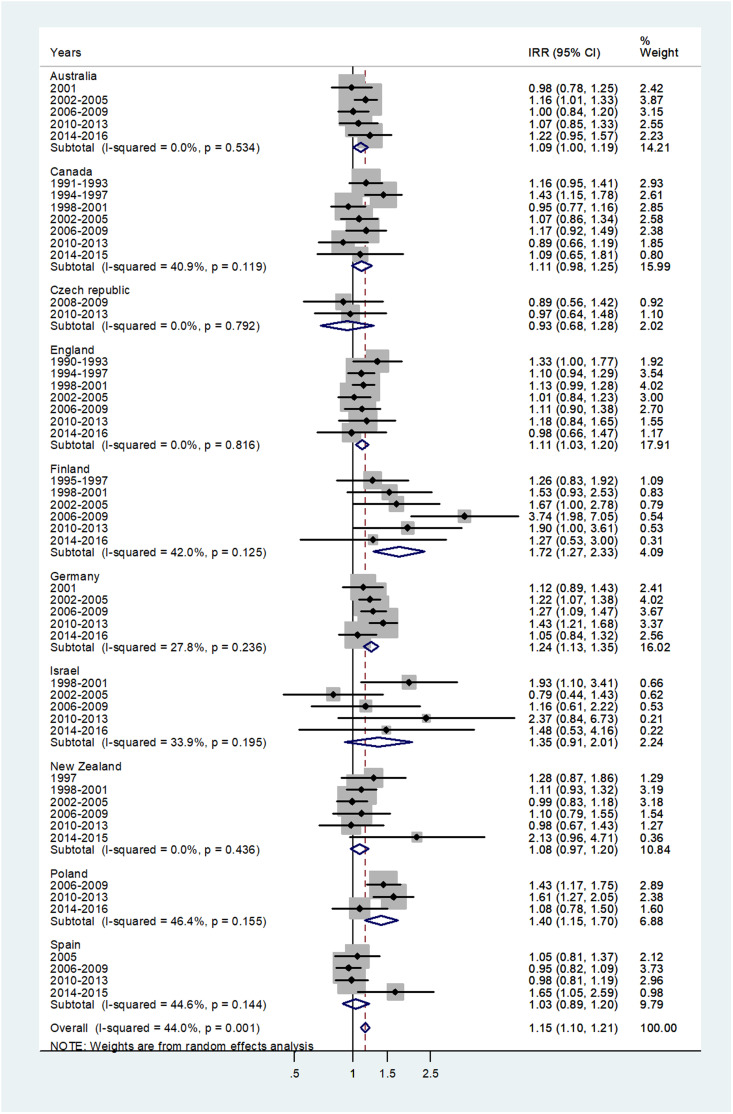
Forest plot of the male to female meningococcal disease IRR for young adults (15–44 or 15–39 years), for 10 countries by time period. CI = 95% confidence interval.

The forest plot for age 15–44 is shown in Figure 5. The pooled IRR = 1.15 (95% CI 1.10–1.21) with *I*^2^ = 44% and ranged from 0.93 for the Czech Republic to 1.72 for Finland.

**Fig. 6.**
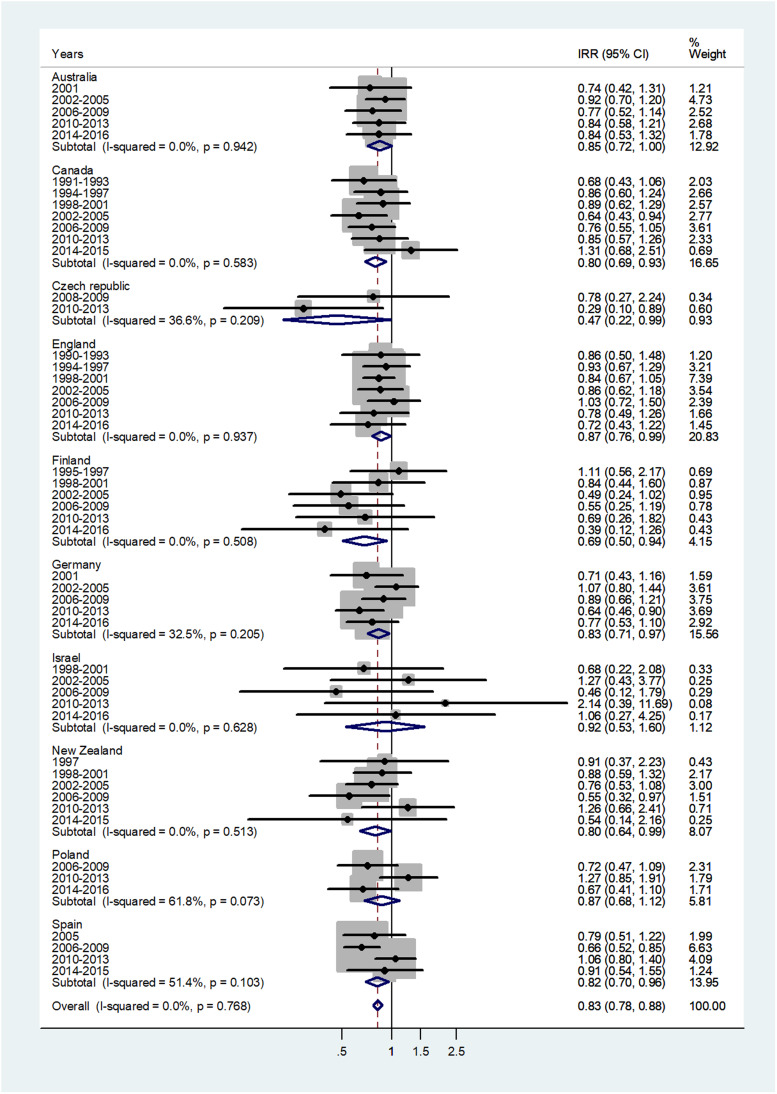
Forest plot of the male to female meningococcal disease IRR for middle age adults (45–64 or 40–59 years), for 10 countries by time period. CI = 95% confidence interval.

The forest plot for age 45–64 is given in Figure 6. The pooled IRR = 0.83 (95% CI 0.78–0.88) with *I*^2^ = 0% and ranged from 0.47 in Czech Republic to 0.92 in Israel.

**Fig. 7.**
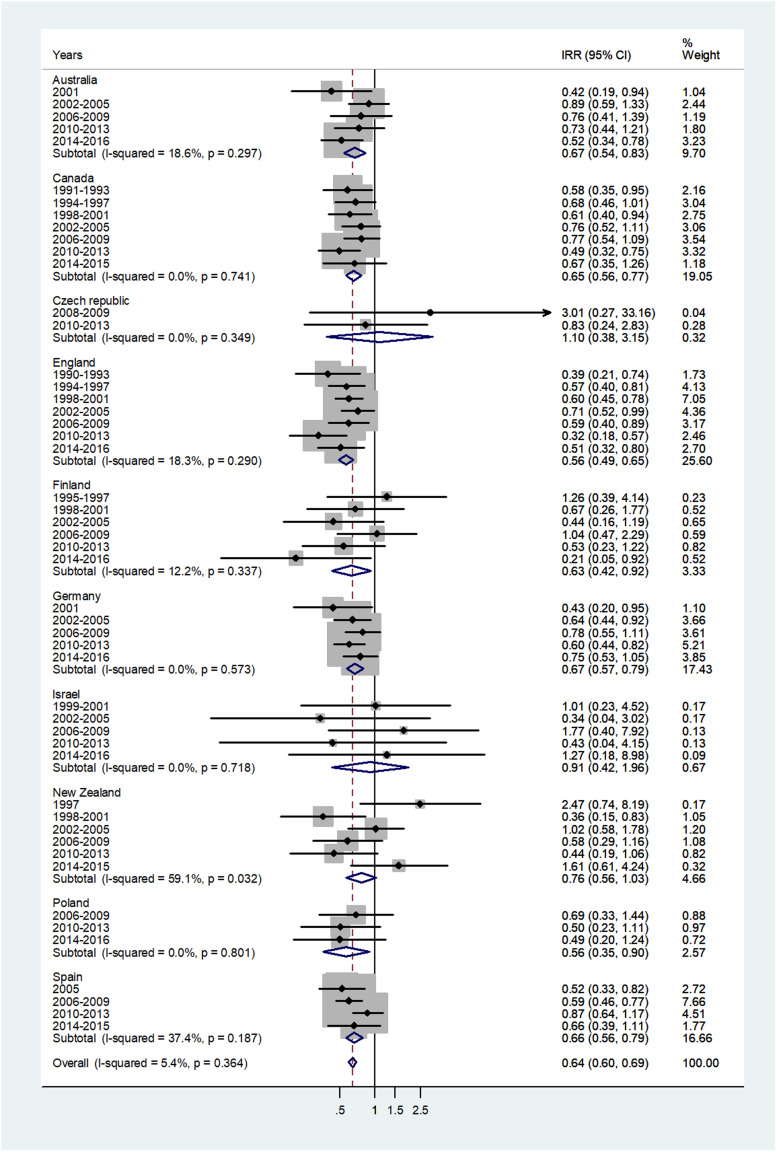
Forest plot of the male to female meningococcal disease IRR for senior adults (60+ or 65+ years), for 10 countries by time period. CI = 95% confidence interval.

The forest plot for age 65+ is given in Figure 7. The overall IRR = 0.64 (95% CI 0.60–0.69) with *I*^2^ = 5.4%, and ranged from 0.56 in England and Poland to 1.10 in Czech Republic.

In order to identify the variables (individual country and year period) that may have an excessive influence on the pooled IRR, we performed leave-one-out sensitivity meta-analysis. After omitting one country or one group of years at a time, the pooled IRRs changed only slightly (Appendix, Supplementary Table S1 and Table S2, respectively). Egger's test *p* value for asymmetry was significant only for late childhood and young adulthood, *P* = 0.069 and *P* = 0.065, respectively. Data are presented in Appendix A, supplementary figure A1 (A−G).

In the meta-regression analyses, age group contributed almost all of the variation in the IRRs. For infants the male IRR was higher than in middle and senior adulthood (*P*<0.0001). The IRRs for early and late childhood were significantly higher than in the older age groups (*P*<0.0001). There was no significant variation between time periods except in age 5−9 where the IRR increased with time (*P* = 0.05).

## Discussion

In the present study of 10 countries, over extended time periods, with the exception of Czech Republic, there were consistently higher meningococcal IR in males, infants, children and young adults. The excess in males ranged from 25%, 24%, 13% and 21% in infants, toddlers, children and young adults. In older adults, the sex ratio was reversed, and females had higher IR. In the age group 45–64, males had a pooled IR 17% lower than females and in the age group 65+, the pooled IR for males was 36% lower than for females. While there were differences in the magnitude of the differences, these findings were remarkably consistent over countries and time periods. There is no clear explanation for the difference findings for the Czech Republic. Their IR were much lower than other countries and consequently less stable.

Previous studies reporting sex differences meningococcal disease are varied in design and it is difficult to assess the differences by age or time period. Higher IR of disease in young males have been reported from population-based studies and case series and case−control studies, for varying age groups and selected time periods. The reports on the older age groups are sparse. In a study conducted in Ireland, the male:female IRRs were 1.29 in those <1 year, 1.50 in the 1–4 group and 0.68 in the over 65 group, with IRRs for all other age groups close to 1 [11]. In studies in Argentina [12] on patients ⩽15 years of age conducted at six paediatric hospitals and on a cohort of 119 patients in Croatia over a 15-year period [13], there were more male than female patients (57% and 58%, respectively). In a study of national surveillance records in Germany between 2001 and 2013, the incidence of meningococcal disease in the age group 1–14 was higher in males [14]. In the Ireland national surveillance data study, 59% of cases with a median age of 5 years (range 0.1–18) were males [15].

The current study has several strengths. We used national population denominators to compute IR from 10 countries with similar lifestyles, developed health systems and social norms. In that sense, they appear to be largely representative of the target population of Europe, North America, Australia/New Zealand and Israel. All have contributed large populations and numbers of cases, over a number of years. Thus, selection bias is unlikely to have a major impact on the findings. Three countries in this study, Germany, Spain and England, routinely immunise infants against meningococcus C. In the sensitivity analysis. However, removing these countries did not affect the sex ratio findings. National data are much less accessible in countries in Africa and Asia, and thus these findings may not be directly applicable to these regions. Although we cannot suggest any reasons why these findings should not apply to such countries, it would be useful to confirm them in studies specifically allowing for the deficiencies in the data. Another possible source of selection bias could be selective care-seeking behaviour or immunisation by sex in infants and young children. However, such practices have not been reported for any of the countries in this study. In adults, there may be some sex differences in the use of health services [43], but again, it is unlikely to be a factor for a disease with such severe clinical manifestations. Information bias could be present in this study due to several factors. First, underreporting could be present, although it should not differ by sex in the countries under study. In addition, underreporting of meningococcal disease is likely to be less than infectious diseases with milder clinical manifestations. The sensitivity and specificity of laboratory diagnosis for meningococcal disease may be heterogeneous, but again should not differ between females and males, and thus be non-differential. If present, it could underestimate the magnitude of the sex IR ratios. The sex differences we observed in reported IR could reflect differences in severity of the disease between the sexes.

As regards possible sex differences in exposure, this seems highly unlikely in infants and young children [44]. This could occur in adults where risk factors include cigarette smoking, men who have sex with men and illicit drug use [45]. In this study, among adults, higher IR were observed in older women. This may be related to greater exposure of women while caring for young children in the home, in daycare centres and in healthcare settings. A study from Ireland, based on national surveillance rates, showed a slight male predominance in IR, but the findings were not broken down by age [11]. In a prospective surveillance of meningococcal meningitis or disease in patients in six paediatric hospital sentinel units in Argentina, 54% of the patients were male [12].

While this study cannot provide information on the mechanisms that could explain the sex differences observed, we can speculate on a number of possibilities. The mechanism underlying the sex differences could occur at the level of colonisation, invasion and/or the immune response. In one study, the prevalence of carriage increased from 4.5% in infants to 23.7% in adults, and then decreased to 7.8% in 50-year olds [6]. Carriage rates in young students have been found to be more common in males in young students [7]. It is possible that cigarette smoking may facilitate the entry of the bacteria through the respiratory tract.

The major mechanism preventing dissemination of *N. meningitidis* from the nasopharynx in the carriage state, is immune lysis induced by serum bactericidal antibody (SBA) [46]. In the first few months of life the infant is protected by maternal antibodies [47]. Natural immunity is acquired through the development of SBA due to exposure to *N. meningitidis*. Sex hormones can act as important modulators of immune response to *N. meningitidis*. Females produce more robust cellular and humoral immune reactions and are more resistant to infections, as compared with males [48]. The male sex hormone testosterone is generally immunosuppressive, while the female sex hormone oestrogen tends to be immunoenhancing [48]. Hormonal differences are present from a very young age with transient sex-specific activation of the hypothalamic−pituitary−gonadal axis in healthy infants [49]. In general, the total oestrogenic bioactivity in pre-pubertal girls is significantly higher than in pre-pubertal boys [50].

The levels of SBA increase with age, and the infant and toddler are most susceptible when the levels are low. The lipid A moiety of lipoooligosaccharide (LOS) on the outer membrane interacts with receptors of the innate immune system [46]. It has been postulated that that variability in lipid A phosphorylation can impact on the innate immune response affecting the clinical outcome of the disease [46]. The extensive release of lipooligosaccharide (LOS) from *N. meningitidis* causes immune cell activation and release of proinflammatory cytokines [51]. The innate immune system expresses pattern recognition receptors (PRRs), which include the Toll-like receptors as the key factor for *N. meningitidis* LOS detection, evoke an intense inflammatory response [52]. All these factors could potentially be affected by sex hormones and explain, at least in part, the excess meningococcal IR observed in young males.

Genetic differences are also responsible for regulation of the immune system and may play a key role in modulating sex differences in the response to infections such as meningococcal disease [8]. Chromosome inactivation, genes escaping X chromosome inactivation, female mosaicism and heterogeneity in X chromosome inactivation patterns all contribute to differences in the immune responses between males and females at cellular and molecular levels [53].

The immune response to *N. meningitidis* is very much dependent on the antibody activation of the complement cascade. Deficiency of the complement cascade increases susceptibility to meningococcal disease [54]. Paradoxically, the complement system has also been shown to contribute to tissue injury [55]. Gaya da Costa M et al. [56] demonstrated significant sex differences in complement levels and functionality in the healthy population.

## Conclusions

This large study has clearly demonstrated a consistent excess IR of meningococcal disease in young males, which is reversed in older adults. The mechanism remains unknown. In young children an interplay between genetic and hormonal factors could be dominant whereas in older people, exposure differences are likely to be more common. Further research on the consistent sex differences in the IR of the meningococcal disease can contribute to a better understanding of the mechanism of the disease.

## Data Availability

The authors confirm that the data supporting the findings of this study are available within the article as Table 1.

## Supplementary material

For supplementary material accompanying this paper visit https://doi.org/10.1017/S0950268820002356. click here to view supplementary material
