# Supplementary material for: A meta-analytic evaluation of sex differences in meningococcal disease incidence rates in 10 countries
Source: Epidemiol Infect. 2020 Oct 2;148:e246. doi: 10.1017/S0950268820002356 (PMC7592104; doi:10.1017/S0950268820002356)
Supplement: Supplementary file 1 [file S0950268820002356sup.zip › S0950268820002356sup001.docx]

**Appendix (Supplementary Table S1 and Table S2)**

**Supplementary Table S1. Sensitivity analysis, by age group and country.**

| **Country removed** | **Age group** | | | | | | |
| --- | --- | --- | --- | --- | --- | --- | --- |
|  | **Infants**  **IRR (CI)** | **Early childhood**  **IRR (CI)** | **Late childhood**  **IRR (CI)** | **Puberty**  **IRR (CI)** | **Young adulthood**  **IRR (CI)** | **Middle adulthood**  **IRR (CI)** | **Senior adulthood**  **IRR (CI)** |
| **Australia** | - | - | 1.13  (1.06-1.19) | 1.2  (1.12-1.29) | 1.18  (1.08-1.29) | 0.82  (0.77-0.88) | 0.63  (0.59-0.68) |
| **Canada** | 1.26  (1.21-1.32) | 1.24  (1.2-1.29) | 1.14  (1.08-1.21) | 1.22  (1.13-1.31) | 1.18  (1.08-1.29) | 0.83  (0.78-0.89) | 0.64  (0.59-0.69) |
| **Czech Republic** | 1.26  (1.21-1.31) | 1.25  (1.21- 1.29) | 1.13  (1.07-1.2) | 1.21  (1.13-1.3) | 1.18  (1.09-1.28) | 0.83  (0.78-0.88) | 0.64  (0.59-0.68) |
| **England** | 1.23  (1.16-1.3) | 1.22  (1.16-1.27) | 1.13  (1.05-1.21) | 1.2  (1.1-1.31) | 1.18  (1.08-1.29) | 0.82  (0.76-0.87) | 0.67  (0.61-0.72) |
| **Finland** | - | - | 1.13  (1.07-1.2) | 1.2  (1.12-1.29) | 1.14  (1.06-1.21) | 0.83  (0.78-0.88) | 0.64  (0.59-0.69) |
| **Germany** | 1.26  (1.21-1.32) | 1.25  (1.21-1.3) | 1.14  (1.08-1.21) | 1.25  (1.16-1.34) | 1.16  (1.07-1.26) | 0.83  (0.77-0.88) | 0.63  (0.58-0.68) |
| **Israel** | 1.25  (1.2-1.31) | 1.24  (1.2-1.29) | 1.12  (1.06-1.19) | 1.21  (1.13-1.29) | 1.16  (1.07-1.26) | 0.83  (0.78-0.88) | 0.64  (0.59-0.68) |
| **New Zealand** | 1.24  (1.19-1.3) | 1.24  (1.19-1.29) | 1.13  (1.06-1.2) | 1.17  (1.09-1.26) | 1.18  (1.09-1.29) | 0.83  (0.78-0.88) | 0.63  (0.59-0.68) |
| **Poland** | 1.27  (1.22-1.33) | 1.25  (1.2-1.29) | 1.14  (1.08-1.21) | 1.21  (1.12-1.29) | 1.14  (1.06-1.23) | 0.82  (0.77-0.88) | 0.64  (0.6-0.69) |
| **Spain** | 1.26  (1.21-1.32) | 1.27  (1.22-1.31) | 1.13  (1.06-1.2) | 1.2  (1.12-1.29) | 1.19  (1.1-1.29) | 0.83  (0.78-0.88) | 0.63  (0.59-0.68) |

IRR = Incidence Rate Ratio; CI = confidence interval.

**Supplementary Table S2. Sensitivity analysis, by age group and years.**

| **Years removed** | **Age group** | | | | | | |
| --- | --- | --- | --- | --- | --- | --- | --- |
|  | **Infants**  **IRR (CI)** | **Early childhood**  **IRR (CI)** | **Late childhood**  **IRR (CI)** | **Puberty**  **IRR (CI)** | **Young adulthood**  **IRR (CI)** | **Middle adulthood**  **IRR (CI)** | **Senior adulthood**  **IRR (CI)** |
| **1990-1993** | 1.26  (1.2-1.31) | 1.24  (1.2-1.29) | 1.14  (1.08-1.21) | 1.21  (1.13-1.3) | 1.14  (1.1-1.18) | 0.83  (0.78-0.88) | 0.66  (0.61-0.71) |
| **1994-1997** | 1.23  (1.18-1.29) | 1.25  (1.2-1.29) | 1.15  (1.08-1.22) | 1.21  (1.13-1.31) | 1.13  (1.1-1.17) | 0.82  (0.77-0.87) | 0.65  (0.6-0.7) |
| **1998-2001** | 1.26  (1.2-1.32) | 1.25  (1.2-1.3) | 1.16  (1.08-1.23) | 1.2  (1.11-1.3) | 1.15  (1.11-1.2) | 0.82  (0.77-0.88) | 0.67  (0.62-0.72) |
| **2002-2005** | 1.26  (1.2-1.32) | 1.23  (1.18-1.27) | 1.12  (1.05-1.19) | 1.2  (1.11-1.3) | 1.15  (1.11-1.2) | 0.82  (0.77-0.88) | 0.64  (0.59-0.69) |
| **2006-2009** | 1.26  (1.2-1.32) | 1.25  (1.21-1.3) | 1.12  (1.05-1.2) | 1.21  (1.12-1.31) | 1.14  (1.1-1.19) | 0.85  (0.79-0.91) | 0.64  (0.59-0.69) |
| **2010-2013** | 1.28  (1.22-1.34) | 1.26  (1.22-1.31) | 1.12  (1.06-1.19) | 1.21  (1.12-1.3) | 1.13  (1.09-1.17) | 0.81  (0.76-0.87) | 0.66  (0.61-0.72) |
| **2014-2017** | 1.25  (1.2-1.31) | 1.24  (1.19-1.28) | 1.13  (1.06-1.19) | 1.2  (1.12-1.29) | 1.14  (1.1-1.18) | 0.83  (0.78-0.88) | 0.66  (0.61-0.71) |

IRR = Incidence Rate Ratio; CI = confidence interval.
