# Supplementary figures and images for: A meta-analytic evaluation of sex differences in meningococcal disease incidence rates in 10 countries
Source: Epidemiol Infect. 2020 Oct 2;148:e246. doi: 10.1017/S0950268820002356 (PMC7592104; doi:10.1017/S0950268820002356)

**Appendix A**

Supplementary Figure A1 (A-G). Funnel plots


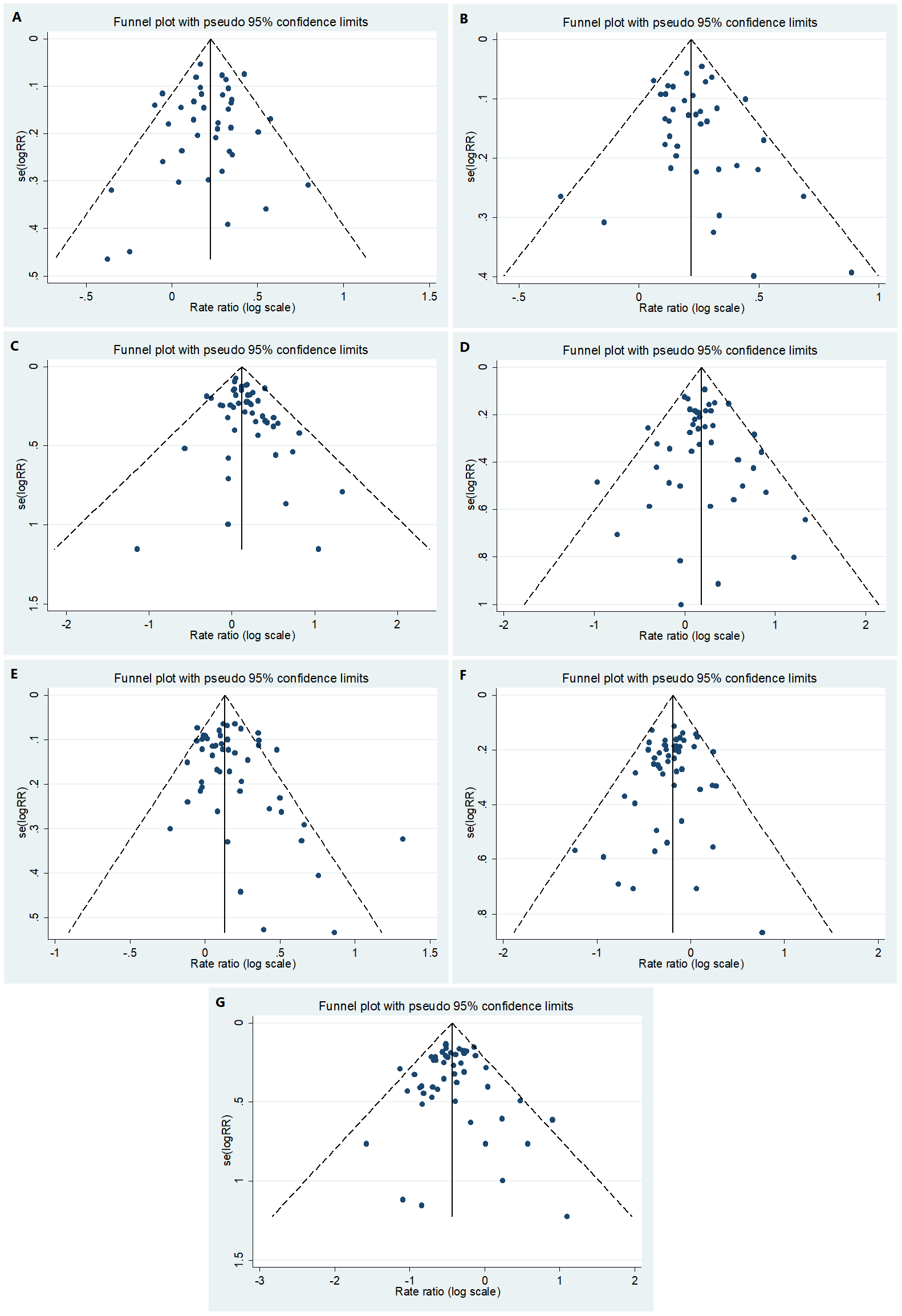

Supplement: Supplementary file 1 [file S0950268820002356sup.zip › S0950268820002356sup002.docx]
